# Association between education and the risk of incident coronary heart disease among middle-aged and older Chinese: the Dongfeng-Tongji Cohort

**DOI:** 10.1038/s41598-017-00880-8

**Published:** 2017-04-10

**Authors:** Hao Wang, Yu Yuan, Lulu Song, Gaokun Qiu, Xuefeng Lai, Liangle Yang, Yang Xiao, Lue Zhou, Handong Yang, Xiulou Li, Chengwei Xu, Xiaomin Zhang, Mei-an He, Tangchun Wu

**Affiliations:** 1 Department of Occupational and Environmental Health, Key Laboratory of Environment and Health, Ministry of Education & Ministry of Environmental Protection, State Key Laboratory of Environmental Health (Incubating), School of Public Health, Tongji Medical College, Huazhong University of Science and Technology, Wuhan, China; 2 Department of Maternal and Child Health, School of Public Health, Tongji Medical College, Huazhong University of Science and Technology, Wuhan, China; 3 Dongfeng Central Hospital, Dongfeng Motor Corporation and Hubei University of Medicine, Shiyan, China

## Abstract

Educational achievement was related to cardiovascular disease in some western populations, but prospective evidence on the relationship between education and the risk of incident coronary heart disease (CHD) in Asians is scarce. We aimed to explore this association and the potential modifying effect of major CHD risk factors related to education in middle-aged and older Chinese adults. We included 18,551 participants with mean age 62.8 years at baseline between September 2008 and June 2010, and followed the population until October 2013. Cox proportional hazard models and log-binomial regression models were used for multivariate analyses. Compared with participants with low education, the adjusted hazard ratio (HR) and 95% confidence interval (95% CI) for incident CHD in those with high education was 0.89 (95% CI: 0.80, 0.98). In addition, such inverse association between education and the risk of incident CHD was more evident among individuals who were more than 60 years, physically active, overweight, and hypertension. Besides, decreases in the relative index of inequality with high education versus low education were from 0.83 to 0.76 for hypertension, from 0.85 to 0.82 for diabetes. In conclusion, education was inversely associated with the risk of incident CHD among middle-aged and older Chinese adults.

## Introduction

Cardiovascular disease (CVD) has become the leading cause of death worldwide^1^. Coronary heart disease (CHD) accounts for the greatest proportion of CVD, and it’s now contributing to large and rising burdens of disease mortality and disability, which increased rapidly in China^2^. Although some important risk factors for CHD are identified, such as overweight, obesity, hypertension, dyslipidemia, and diabetes, efforts are needed to better understand other risk factors of CHD and to develop prevention strategies.

Socioeconomic status (SES) is the root of many inequalities in health^3^. Social inequalities in risk factors account for more than half of inequalities in CVD^4^. SES is usually measured by education, income, occupation, etc. The relationship between these SES measures and health outcome was the strongest for education^5^. As the most basic component of SES, education is prior to occupation and income and stable throughout life after young adulthood^6^. Low education is associated with large increases in CHD risk among men and women in high-income countries^7–12^, whereas studies on the effects of education on CHD risk in low-income and middle-income countries are more scarce^13^. In addition, most of these studies focused on the association between education and mortality or risk factors of CHD^9, 12^. Accumulating data showed that higher education was inversely associated with the mortality or risk factors of CHD^7, 8, 13, 14^, but only a few studies have investigated the relationship between education and the risk of incident CHD. Furthermore, education is also known to be associated with other important CHD risk factors, such as hypertension, hyperlipidemia, overweight and diabetes^15, 16^. However, it remains unclear whether such risk factors would modify the association between education and the risk of incident CHD.

Therefore, we aimed to (1) verify the association between education and the risk of incident CHD, and (2) investigate whether the relationship between education and the risk of incident CHD was modified by different characteristics or health status, as well as the underlying changes of CHD risk factors in China.

## Results

### Baseline characteristics of participants

Baseline characteristics of the 18,551 participants (8,164 men and 10,387 women) are shown in Table 1. Of all the participants, individuals with high education were more likely to be married and physically active, had lower mean values of age, body mass index (BMI) and waist circumference, consumed vegetables and fruits more frequently, and had lower prevalence of hypertension and diabetes compared with those with low education. Lower rates of smoking and alcohol drinking were also observed in participants with high education.

**Table 1:** Baseline characteristics of the study subjects according to the type of educational levels.

Variables	Low education (≤8 years)	High education (≥9 years)	*P*
Sample size, n	12264	6287	<0.01
Mean age, year	63.29 ± 7.28	61.80 ± 8.30	<0.01
Male, n (%)	5253 (42.83)	2911 (46.30)	<0.01
Married, n (%)	10942 (89.43)	5754 (91.71)	<0.01
BMI, kg/m^2^	24.54 ± 3.44	24.00 ± 3.20	<0.01
Waist circumference, cm	83.34 ± 9.47	81.56 ± 9.22	<0.01
Married, n (%)	10942 (89.43)	5754 (91.71)	<0.01
Current Smokers, n (%)	2443 (20.04)	1011 (16.21)	<0.01
Current alcohol drinker, n (%)	2877 (23.49)	1291 (20.55)	<0.01
Physical activity, n (%)	10834 (88.34)	5647 (89.82)	<0.01
Fruit and vegetable intake, n (%)			
Fruit (≥1 time/day)	6253 (50.99)	3865 (61.48)	<0.01
Vegetable (≥1 time/day)	11674 (95.19)	6002 (95.47)	0.21
Stress, n (%)	5459 (44.51)	2822 (44.89)	0.32
Overweight, n (%)	6726 (54.86)	3011 (47.92)	<0.01
Hypertension, n (%)	6095 (49.70)	2717 (43.22)	<0.01
Hyperlipidemia, n (%)	5607 (45.72)	2950 (46.92)	0.06
Diabetes, n (%)	2112 (17.22)	908 (14.44)	<0.01
Family history of CHD, n (%)	234 (2.72)	492 (7.83)	<0.01

Abbreviation: CHD, coronary heart disease; BMI, body mass index.

Data are mean ± standard deviation for continuous variables and number (%) for categorical variables.

*P* values were calculated using student’s t-test for continuous variables and chi-square test for categorical variables.

### Association between education and the risk of incident CHD

Table 2 shows the five years cumulative CHD incidence rates and the adjusted hazard ratios (HRs) of the risk of incident CHD associated with education. In age-adjusted analysis, higher education was associated with a lower risk of CHD incidence in all participants (HR: 0.87; 95% confidence interval (CI): 0.79, 0.96). After further adjustment for smoking status, drinking status, physical activity, marital status, diet frequency categories and stress, the association did not materially change (HR: 0.87; 95% CI: 0.79, 0.96). In the final multivariate adjusted model, the association between education and CHD incidence was slightly attenuated (HR: 0.89; 95% CI: 0.79, 0.98) with additionally adjustment for BMI, waist circumference, hypertension, hyperlipidemia, diabetes and family history of CHD. Beside, higher education was independently associated with the lower risk of non-fatal CHD (HR: 0.90; 95% CI: 0.81, 0.99, Supplementary Table S1). No significant association was observed between education and the risk of fatal CHD (HR: 0.70; 95% CI: 0.43, 1.14, Supplementary Table S1), which might be explained as the small sample size of fatal events limited the statistical power. Furthermore, education was also inversely associated with all-cause mortality among all the participants (HR: 0.78; 95% CI: 0.69, 0.88, Supplementary Table S2).

**Table 2:** HRs and 95% CIs for CHD incidence according to different educational levels.

Model	Low education (≤8 years)	High education (≥9 years)
Case, person (%)	1323/12264 (10.79)	578/6287 (9.19)
Model 1	Reference	0.87 (0.79, 0.96)
Model 2	Reference	0.87 (0.79, 0.96)
Model 3	Reference	0.89 (0.80–0.98)

Model 1 adjusted for age.

Model 2 adjusted for model 1 plus smoking, drinking, physical activity, marital status, stress, fruit intake, and vegetable intake.

Model 3 adjusted for model 2 plus BMI, waist circumference, hypertension, hyperlipidemia, diabetes, and family history of CHD.

### Subgroup analyses

**Figure 1:**
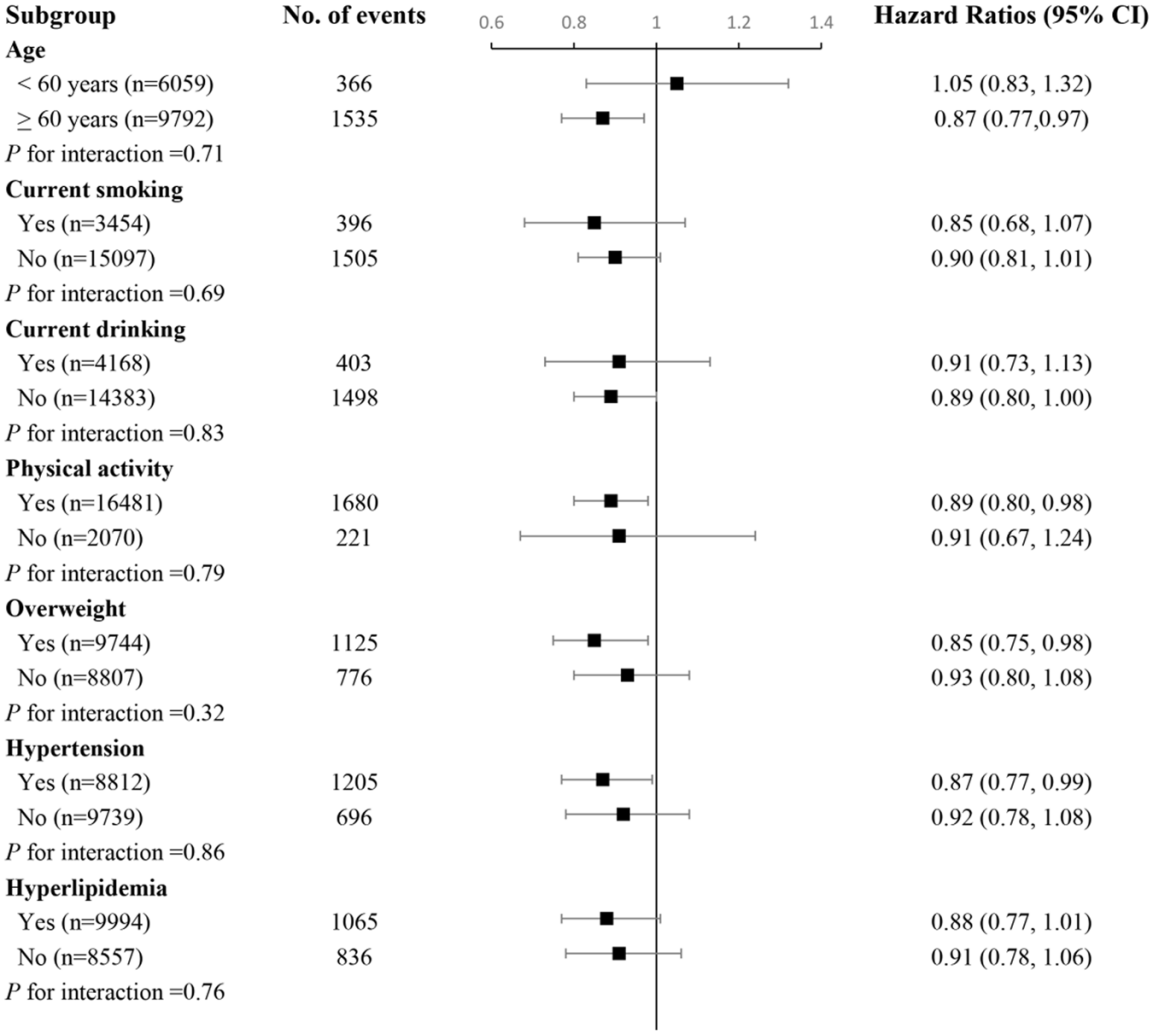
Subgroup analysis of association between education and the risk of incident CHD according to potential risk factors. All covariates were age, smoking, drinking, physical activity, marital status, stress, fruit intake, vegetable intake, BMI, waist circumference, hypertension, hyperlipidemia, diabetes, and family history of CHD. Each group adjusted for the other covariates except itself. The reference group was low education. Horizontal lines represent 95% confidence intervals.

We further performed stratified analysis according to age, current smoking, current drinking, physical activity, overweight, hypertension, and hyperlipidemia (Fig. 1). No significant interactions between education and these characteristics on risk of CHD were detected (all *P* for interaction >0.05). However, the inverse association between education and risk of incident CHD was more pronounced in participants who were older than 60 years, physically active, overweight and hypertension.

### Relative index of inequalities (RIIs) of CHD risk factors from 2008 to 2013

Table 3 manifests some of the changes in CHD risk factors in the two different educational levels from 2008 to 2013. The age-standardized prevalence of hypertension, hyperlipidemia and diabetes worsened, but smoking, alcohol and physical activity improved during the follow years both in participants with low and high education. The RII is used to compare the degree of inequality between educational classes at 2008 and 2013. The closer the RII value is to 1.00, the smaller the disparities between individuals with higher and lower education. A negative RII value indicates that the CHD risk factors prevalence of the individuals with higher education is lower than that of the individuals with lower education. Decreases in the RII from 0.83 (95% CI: 0.78, 0.88) to 0.76 (95% CI: 0.71, 0.81) for hypertension, from 1.07 (95% CI: 1.01, 1.14) to 1.06 (95% CI: 1.00, 1.13) for hyperlipidemia, from 0.85 (95% CI: 0.78, 0.93) to 0.82 (95% CI: 0.76, 0.89) for diabetes. Increases in the RII for use of anti-hyperlipidemia drug were from 1.13 (95% CI: 1.01, 1.27) in 2008 to 1.25 (95% CI: 1.13, 1.38) in 2013, physical activity from 1.22 (95% CI: 1.10, 1.35) to 1.31 (95% CI: 1.17, 1.45). The above changes for the RII were not statistically significant.

**Table 3:** Change in coronary heart disease risk factors by educational level, with comparison of RII.

Risk factors	Year	Low education	High education	RII (95% CI)	*P* for RII change^*^
Current smokers, %	2008	19.83	16.56	0.80 (0.74, 0.87)	0.956
	2013	15.50	13.26	0.83 (0.75, 0.91)	
Alcohol drinking, %	2008	23.32	20.82	0.86 (0.80, 0.93)	0.375
	2013	24.23	22.31	0.89 (0.83, 0.97)	
Physical activity, %	2008	88.17	90.09	1.22 (1.10, 1.35)	0.879
	2013	88.74	91.14	1.31 (1.17, 1.45)	
Overweight, %	2008	54.70	48.22	0.77 (0.73, 0.82)	0.977
	2013	53.49	47.31	0.77 (0.73, 0.83)	
Hypertension, %	2008	49.03	44.47	0.83 (0.78, 0.88)	0.073
	2013	64.53	58.39	0.76 (0.71, 0.81)	
Hyperlipidemia, %	2008	45.52	47.31	1.07 (1.01, 1.14)	0.551
	2013	47.64	49.31	1.06 (1.00, 1.13)	
Diabetes, %	2008	16.99	14.84	0.85 (0.78, 0.93)	0.729
	2013	20.73	17.67	0.82 (0.76, 0.89)	
Hypotensive drug, %	2008	50.73	55.65	1.22 (1.11, 1.34)	0.249
	2013	52.94	55.27	1.10 (1.01, 1.19)	
Anti-hyperlipidemia drug, %	2008	17.62	19.43	1.13 (1.01, 1.27)	0.169
	2013	24.99	29.24	1.25 (1.13, 1.38)	
Hypoglycemic drug, %	2008	46.82	50.46	1.16 (0.99, 1.35)	0.455
	2013	52.54	53.56	1.04 (0.90, 1.21)	

Abbreviation: RII, relative index of inequality. Variables were presented as age-standardized prevalence rate.

^*^Test for change in inequality between 2008 and 2013.

## Discussion

To the best of our knowledge, this is the first prospective study to examine the association between education and the risk of incident CHD in Chinese population. The findings from this study indicated that higher education was independently associated with a reduced risk of incident CHD, even after adjustment for a variety of potential confounders. Such relationship was more obvious among participants who were more than 60 years, physically active, overweight and hypertension. The inequalities of hypertension and diabetes increased between low and high education during the follow-up years.

Some epidemiological studies have investigated the association between SES and CHD, but the measures of SES in those studies were different, such as occupation, social support, neighborhood socioeconomic environment, and life-course socioeconomic position. For instance, the socioeconomic indicator in the Whitehall II study was occupational class^17^, which was complex and changeable. A cohort study in Sweden reported that neighborhood socioeconomic environment was associated with the incidence of CHD^18^. Loucks *et al*. also reported a significant inverse association between life-course socioeconomic position and incidence of CHD^19^. Additionally, several studies examined the relationship between education and mortality from CHD^7^, however, the endpoint was not CHD incidence. Because of the different measures of SES and endpoint, these studies may not easily be compared. Besides, China has the examination-oriented education pattern^20^, socialist system, distinct cultures and values, which are different from western countries^21^. The upper classes in China are more likely to be married, and have better interpersonal relations, stronger power and lower stress^22–24^.

A population-based prospective study of 5,084 participants aged between 35 and 74 reported that low education was associated with high risk of incident CHD in women after adjustment for potential confounders^9^. In addition, a study from the United States indicated an inverse relationship between education and risk of CHD^12^. These studies supported the finding of the present study that education might be inversely associated with the risk of incident CHD. However, some potential confounders were not fully adjusted in these studies, such as diet, physical activity, stress and family history of CHD, which might mediate the relationship of education with the risk of incident CHD. In our study, we have adjusted for diet, physical activity, and family history of CHD, which did not alter the significant association between education and CHD incidence.

Although the underlying mechanisms between education and CHD were unclear, several plausible mechanisms might be involved in the association. Firstly, it is widely recognized that education can lead to improved health by increasing health knowledge and healthy lifestyle behaviors^25^, which was found to be inversely associated with all-cause mortality, especially for CHD mortality^26–28^. Secondly, strong and consistent evidence indicated that parental SES, childhood and early-life factors would contribute to the elevated risk of incident CHD^29, 30^. Almost all the participants in our study were born before 1970s, when high level of education was extravagant. Thus, participants with higher education often had a higher socioeconomic gradient during their childhood, which decreased the risk of incident CHD through their lifetimes^31^. Thirdly, education also plays an important role in CVD by shaping employment opportunities, which are major determinants of economic resources. Individuals with high education tended to experience lower rates of unemployment, which is strongly associated with CVD^32^. Finally, educational inequalities also exist in financial and physical access to health care, and health-care use and quality. These factors might lead to inequalities in the diagnosis and treatment of predisposing factors of CHD, and the screening of CHD^4^. In the present study, use of medication for secondary prevention after hypertension, hyperlipidemia and diabetes was higher in high educational patients than low educational patients.

In the stratified analysis, we found that the inverse association between education and the risk of incident CHD risk was more pronounced in individuals who were more than 60 years old, physically active, overweight, and hypertension, although no interaction was observed. Pekka *et al*. previously found that the inverse relationship between education and CHD was more pronounced in elderly people^33^, which was in line with our finding. According to the theory of cumulative advantage of education on health, education differentials in CHD incidence would be smaller at younger ages than at older ages^34^. Moreover, higher educational individuals with overweight or hypertension are more likely to improve their unhealthy behaviors or choose better medical care to reduce the risk of incident CHD^35^.

Most of the previous studies on the association between education and CHD did not include the corresponding changes in CHD risk factors. In contrast, we found that high education was related to favorable changes at 5-year follow-up compared with low education in the RIIs of hypertension and diabetes. Inequalities in the prevalence of hypertension and diabetes have increased over time between low and high education. These changes might be caused by different lifestyles (diet, physical activity, smoking, alcohol drinking) and health care (usage of drug) between low and high education^36^. In addition, above risk factors are common predisposing factors for CHD. It is widely acknowledged that hypertension and diabetes are strong risk factors for development of CHD, and may link education to incident CHD^37^.

The strengths of our study are the relatively large sample size, the prospective design, validated CHD ascertainment, and the ability to adjust for a large number of potential confounders. There are also some limitations that should be taken into consideration. Firstly, we did not collect the information on income, which is also an important indicator of SES. However, education, the facet of socioeconomic status, has been reported as more determinant of health status, particularly cardiovascular conditions. It is the only indicator that remains relatively consistent through adult life. Secondly, education was only classified into two groups, including high (≥9 years) and low (≤8 years) educational level. In participants’ studenthood in China, only individuals with economic strength and strong learning ability had access to receiving high school education and above (≥9 years), which were considered as luxuries and high degrees. Thus, we select 8 years of education as the cut off.

In conclusion, education was inversely associated with the risk of incident CHD in China, and this association was modified by different CHD risk factors. To our knowledge, this is the first study to investigate the association between education and the risk of incident CHD in China. Our study findings have implication for targeted preventive care of CHD. Taking targeted measures to prevent CHD for participants with low education or improving educational inequalities may reduce the disease burden caused by CHD or the health inequalities caused by social inequalities.

## Method

### Study population

Data for the present analyses were based on the Dongfeng-Tongji Cohort Study, and detailed information on the design and method of this study has been reported previously^38^. In brief, a total of 27,009 retired employees of the Dongfeng Motor Corporation completed baseline questionnaires, took medical examinations, and provided fasting blood samples between September 2008 and June 2010. The follow-up survey was conducted in 2013. Among 25,978 individuals (96.2% of those at baseline) who completed the first follow-up, we excluded individuals with self-reported CHD, abnormal electrocardiogram, stroke, or cancer at baseline (n = 6,712), as well as those with missing data on education and other covariates (n = 715), resulting in a final study sample of 18,551 individuals. Informed consent was provided by every participant. Ethics approvals were obtained from the Ethics and Human Subject committee of Tongji Medical College, Huazhong University of Science and Technology and Dongfeng General Hospital. All the methods in the present study were carried out in accordance with the approved guidelines.

### Baseline assessment

Semi-structured questionnaires were used to collected baseline data by trained interviewers. Questionnaires included information on socio-demographic characteristics (e.g., age, sex and marital status), lifestyle factors (e.g., smoking status, alcohol drinking status, diet, and physical activity), self-reported personal and family medical history, medications, stress status and occupational history. Physical activity was defined as those who took exercise more than 20 minutes per day and at least 3 times per week over half of a year^39^. Dietary factors included vegetable and fruit intake (rarely, or at least once per day)^40^. BMI was calculated as weight in kilograms divided by height squared in meters and rounded to the nearest tenth. The individuals with BMI equal to or over 24 kg/m^2^ were categorized into overweight group, and others were categorized into normal group^41^. Hypertension was defined as a systolic blood pressure (SBP) ≥140 mm Hg, or a diastolic blood pressure (DBP) ≥90 mm Hg, or current use of antihypertensive medication, or self-reported physician diagnosis of hypertension^42^. High blood cholesterol was defined as total serum cholesterol (CHOL) >5.72 mmol/L or triglycerides (TG) >1.70 mmol/L or use of lipid-lowering medication, or self-reported physician diagnosis of hyperlipidemia^43^. Diabetes mellitus was considered as a fasting glucose (GLU) level >7.0 mmol/L or receiving anti-diabetic medications, or self-reported physician diagnosis of diabetes^44^.

### Classification of education

Information on the educational attainment was assessed via questionnaire. We derived a two-group classification of education: low education and high education. Low education (8≤ years) included illiteracy, primary school and junior high school; high education (≥9 years) included high school, bachelor and master or above.

### Ascertainment of CHD incidence

All participants were covered by the Dongfeng health-care service system with specific identification number to retrieve medication information. Incident CHD cases were confirmed by review of medical insurance documentation, hospital records of the Dongfeng Central hospital and other affiliated hospitals, and death certificates. Among participants with suspicion of CHD, all the medical information were retrieved and discussed by a group of physicians in the Dongfeng Central hospital. The incidence caused by CHD included first occurrence of non-fatal myocardial infarction (non-fatal MI), fatal CHD, stable angina (SA), unstable angina (UA), and coronary revascularization, which were defined based on the diagnosis criteria of the World Health Organization by searching clinical symptoms, myocardial enzyme, ECG and coronary angiography (at least, the stenosis of one major coronary artery >50%)^45, 46^. The cases of CHD were divided into fatal CHD and non-fatal CHD according to the case definition^46^. We define fatal CHD events if the participant died with coronary heart disease as an underlying cause, which were ascertained by death certificates with International Classification of Diseases (ICD) codes (ICD-9 410-414 and ICD-10 I20-I25). Non-fatal events included non-fatal MI, stable or unstable angina, and coronary revascularization.

### Statistical Analysis

Distributions of baseline characteristics of the study population were compared using One-way analysis of variance (ANOVA) for continuous variables and Chi-square tests for categorical variables. Continuous variables were described as mean ± standard deviation and categorical variables were presented as proportion. Cox proportional hazard models were used to evaluate the association between education and the risk of incident CHD calculated with HR and 95% CI, after adjustment for potential confounders. The low education was regarded as the reference group. We also evaluated the association of education with fatal and non-fatal CHD separately. We fitted three proportional hazard models. In Model 1 we only adjusted for age. Model 2 was adjusted for age, smoking status (current, former, never), alcohol drinking status (current, former, never), physical activity (yes or no), diet frequency categories (including vegetables and fruits), and stress (yes or no). Model 3 was further adjusted for BMI, waist circumference, hypertension, diabetes, hyperlipidemia, and family history of CHD. We also conducted analyses in different subgroups stratified by age, smoking status, drinking status, physical activity, overweight, hypertension and hyperlipidemia. Besides, the association between education and all-cause mortality was explored with age-adjusted cox proportional hazard models. Furthermore, age-standardized prevalence rate was used as the absolute measures, while RII was used as the relative effect size. Comparison of the degree of inequality in risk factors was carried out by calculating the RII, which is interpreted as an odds ratio, and 95% CI. The RII takes into account both the population size and the relative socioeconomic position of the groups. It was estimated by employing a log-binomial regression^47^. Analyses were performed using SAS version 9.4 (SAS institute Inc., Cary, NC). All statistical tests were two-tailed and considered to be significant at *P* value less than 0.05.

## Electronic supplementary material

Supplementary Information
