# Supplementary material for: Association between education and the risk of incident coronary heart disease among middle-aged and older Chinese: the Dongfeng-Tongji Cohort
Source: Sci Rep. 2017 Apr 10;7:776. doi: 10.1038/s41598-017-00880-8 (PMC5429684; doi:10.1038/s41598-017-00880-8)
Supplement: Supplementary file 1 — Supplementary Information [file 41598_2017_880_MOESM1_ESM.pdf]

Supplementary Information

Association between education and the risk of incident coronary heart disease among middle-aged and older Chinese: the Dongfeng-Tongji Cohort

Hao Wang, Yu Yuan, Lulu Song, Gaokun Qiu, Xuefeng Lai, Liangle Yang, Yang Xiao, Lue Zhou, Handong Yang, Xiulou Li, Chengwei Xu, Xiaomin Zhang, Mei-an He, Tangchun Wu

Supplementary Table S1 HRs and 95% CIs for fatal and nonfatal CHD according to different educational level

Model	Low education (≤ 8 years)	High education (≥ 9 years)
**Fatal CHD**		
Case, person (%)	66/11007 (0.60)	22/5731 (0.38)
Model 1	Reference	0.68 (0.42, 1.10)
Model 2	Reference	0.67 (0.41, 1.10)
Model 3	Reference	0.70 (0.43, 1.14)
**Non-fatal CHD**		
Case, person (%)	1257/12198 (10.30)	556/6265 (8.87)
Model 1	Reference	0.87 (0.79, 0.97)
Model 2	Reference	0.88 (0.79, 0.97)
Model 3	Reference	0.90 (0.81, 0.99)

Model 1 adjusted for age

Model 2 adjusted for model 1 plus smoking, drinking, physical activity, marital status, stress, fruit intake, and vegetable intake.

Model 3 adjusted for model 2 plus BMI, waist circumference, hypertension, hyperlipidemia, diabetes, and family history of CHD.

Supplementary Table S2 Age-adjusted HRs and 95% CIs for all-cause mortality according to different educational level

	Low education (≤ 8 years)	High education (≥ 9 years)
Case, person (%)	993/17465 (5.69)	370/ 9322 (3.97)
Hazard ratio	Reference	0.78 (0.69,0.88)
